# NAD-Independent L-Lactate Dehydrogenase Is Required for L-Lactate Utilization in *Pseudomonas stutzeri* SDM

**DOI:** 10.1371/journal.pone.0036519

**Published:** 2012-05-04

**Authors:** Chao Gao, Tianyi Jiang, Peipei Dou, Cuiqing Ma, Lixiang Li, Jian Kong, Ping Xu

**Affiliations:** 1 State Key Laboratory of Microbial Technology, Shandong University, Jinan, People's Republic of China; 2 State Key Laboratory of Microbial Metabolism and School of Life Sciences and Biotechnology, Shanghai Jiao Tong University, Shanghai, People's Republic of China; Institut Pasteur Paris, France

## Abstract

**Background:**

Various *Pseudomonas* strains can use l-lactate as their sole carbon source for growth. However, the l-lactate-utilizing enzymes in *Pseudomonas* have never been identified and further studied.

**Methodology/Principal Findings:**

An NAD-independent l-lactate dehydrogenase (l-iLDH) was purified from the membrane fraction of *Pseudomonas stutzeri* SDM. The enzyme catalyzes the oxidation of l-lactate to pyruvate by using FMN as cofactor. After cloning its encoding gene (*lldD*), l-iLDH was successfully expressed, purified from a recombinant *Escherichia coli* strain, and characterized. An *lldD* mutant of *P. stutzeri* SDM was constructed by gene knockout technology. This mutant was unable to grow on l-lactate, but retained the ability to grow on pyruvate.

**Conclusions/Significance:**

It is proposed that l-iLDH plays an indispensable function in *Pseudomonas* l-lactate utilization by catalyzing the conversion of l-lactate into pyruvate.

## Introduction

*Pseudomonas* strains are Gram-negative rod-shaped bacteria commonly found in soil, water, and plant and animal tissues [1], [2]. They have very simple nutritional requirements and can grow well with a single organic molecule such as lactate as the sole carbon and energy source. In the lactate utilization processes of *Pseudomonas aeruginosa*, *P. putida*, and *P. stutzeri*, lactate is first converted to pyruvate, and then metabolized through the tricarboxylic acid cycle [3]–[5]. However, the enzymes responsible for the oxidation of lactate to pyruvate in these *Pseudomonas* strains have not been identified and further studied.

NAD-Independent l-lactate dehydrogenases (l-iLDHs), which catalyze the oxidation of l-lactate to pyruvate by an FMN-dependent mechanism, are widely distributed among bacteria, yeast, and protists [6]–[8]. These enzymes have been studied extensively in *Escherichia coli* and *Saccharomyces cerevisiae* [7], [9]–[11]. In *E. coli*, l-iLDH is a peripheral membrane protein, while in *S. cerevisiae*, it is located in the mitochondrial intermembrane space [6], [10], [11]. l-iLDHs in *E. coli* and *S. cerevisiae* have been purified and further characterized [6], [10], [11]. They catalyze the oxidation of l-lactate to pyruvate through the respiratory electron transport chain *in vivo* and allow these strains to grow well in medium containing l-lactate as the sole carbon source [6], [9], [12].

Previous works have also confirmed the presence of l-iLDHs in *P. aeruginosa*, *P. putida*, and *P. stutzeri* [3]–[5], [13], [14]. However, l-iLDHs are only induced when these *Pseudomonas* strains are grown aerobically with l-lactate as the carbon source [3]–[5], [13]. Based on this observation, involvement of l-iLDHs in *Pseudomonas* l-lactate utilization has been speculated [3]–[5], [13]. As in *E. coli*, l-iLDHs in *Pseudomonas* strains are membrane-bound proteins [13], [14] and it is difficult to purify them. Therefore, there is a lack of information on the properties and functions of l-iLDHs in *Pseudomonas* strains.

In this study, a membrane-bound l-iLDH from *P. stutzeri* strain SDM was purified, and its encoding gene, *lldD*, was cloned, expressed, and characterized. A mutant of strain SDM was constructed by knockout of the *lldD* gene. The mutant was unable to grow with l-lactate as the sole carbon source, providing evidence for an indispensable role of l-iLDH in l-lactate utilization in this *Pseudomonas* strain.

## Results and Discussion

### Purification of l-iLDH

**Figure 1:**
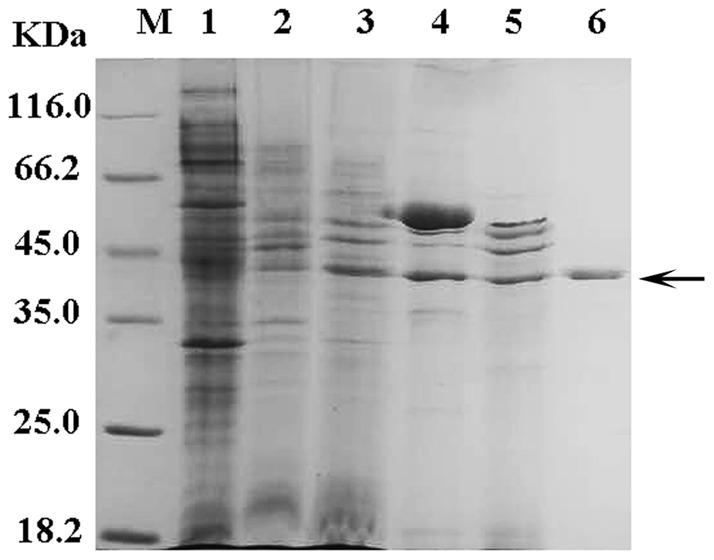
SDS-PAGE analysis of the purified l-iLDH from *P. stutzeri* SDM. Lane M, molecular weight markers; lane 1, crude extract; lane 2, membrane fraction; lane 3, Triton X-100 extract; lane 4, active fraction after ammonium sulfate precipitation; lane 5, active fraction after DEAE Sepharose fast flow column-based extraction; lane 6, the purified l-iLDH in *P. stutzeri* SDM. Protein bands were visualized by Coomassie Blue staining. The arrow indicates the purified l-iLDH in *P. stutzeri* SDM.

Purification of membrane-bound l-iLDHs in *Pseudomonas* species has never been reported. In this study, the membrane-bound l-iLDH in *P. stutzeri* SDM was solubilized with Triton X-100 and purified. The results of a typical purification procedure are summarized in Figure 1 and Table 1. The specific activity at the final step was 83.0 U mg^−1^ of protein, which was a 364.5-fold increase over that of crude cell extract. The molecular mass of l-iLDH was found to be 42.8±0.6 kDa (Figure 1), using sodium dodecyl sulfate-polyacrylamide gel electrophoresis (SDS-PAGE) with 12.5% gel.

**Table 1:** Purification of l-iLDH from *P. stutzeri* SDM.

Purification step	Specific activity (U mg^−1^)	Purification (fold)	Yield (%)
Crude cell extract	0.2	1	100
Membrane fraction	0.7	2.9	61.0
Triton X-100 extract	2.5	11.1	48.2
Ammonium sulfate fractionation	6.5	28.3	21.5
DEAE-Sepharose	12.0	52.7	8.1
Source 30Q	83.0	364.5	2.6

### Cofactor analysis of l-iLDH

**Figure 2:**
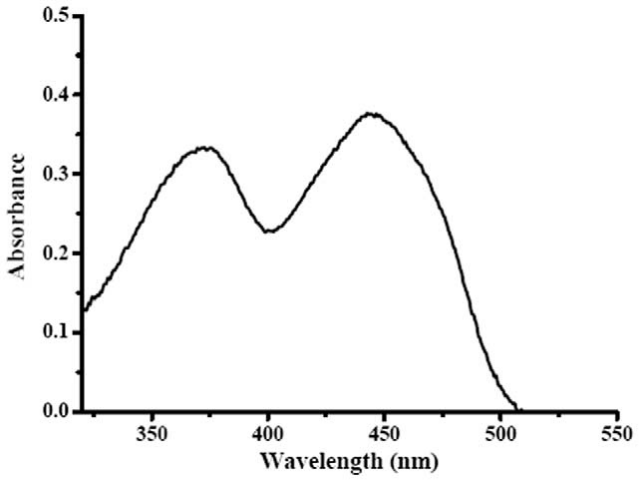
UV-visible absorption spectrum of l-iLDH in *P. stutzeri* SDM. The UV-visible spectrum was recorded with a spectral resolution of 0.5 nm. The absorption spectrum of l-iLDH has two peaks, at around 460 nm and 380 nm, suggesting the presence of flavin in purified l-iLDH.

The purified l-iLDH has an intense yellow color. The absorption spectrum of l-iLDH in the visible region has peaks at around 460 nm and 380 nm, suggesting that l-iLDH is a flavoprotein (Figure 2). The flavin was released from the protein by boiling. The released flavin was confirmed to be FMN based on its identical migration with authentic FMN on high-performance liquid chromatography (HPLC) (Figure S1). Analysis of the flavin content of several preparations of homogeneous enzyme identified the ratio of l-iLDH to FMN as 1.03. Therefore, the native enzyme contains one FMN per subunit (Figure S2).

### Sequence analysis of l-iLDH

Edman degradation analysis revealed that the N-terminal amino acid sequence of l-iLDH from *P. stutzeri* SDM is MIISASTDYRAAA. Two internal peptides were obtained by electrospray ionization-tandem mass spectrometry (ESI-MS/MS) of the peptides resulting from trypsin digestion (AAGVTTLVFTVDMPTPGAR and DAVTFGADGIIVSNHGGR). The three peptides exhibit high similarity to the putative FMN-dependent l-lactate dehydrogenases in *P. putida* KT2440 (AAN70308.1) [15], *P. aeruginosa* PA01 (AAG08157.1) [16], and *P. entomophila* L48 (CAK13685.1) [17]. The l-iLDH coding gene (denominated as *lldD* in this work) was cloned from the genome of *P. stutzeri* SDM by PCR. Its sequence also exhibited high similarity to the genes encoding the putative l-lactate dehydrogenases from *P. putida* KT2440 [15], *P. aeruginosa* PA01 [16], and *P. entomophila* [17]. These results implied that the putative proteins might also exhibit l-iLDH activity in these *Pseudomonas* strains.

**Figure 3:**
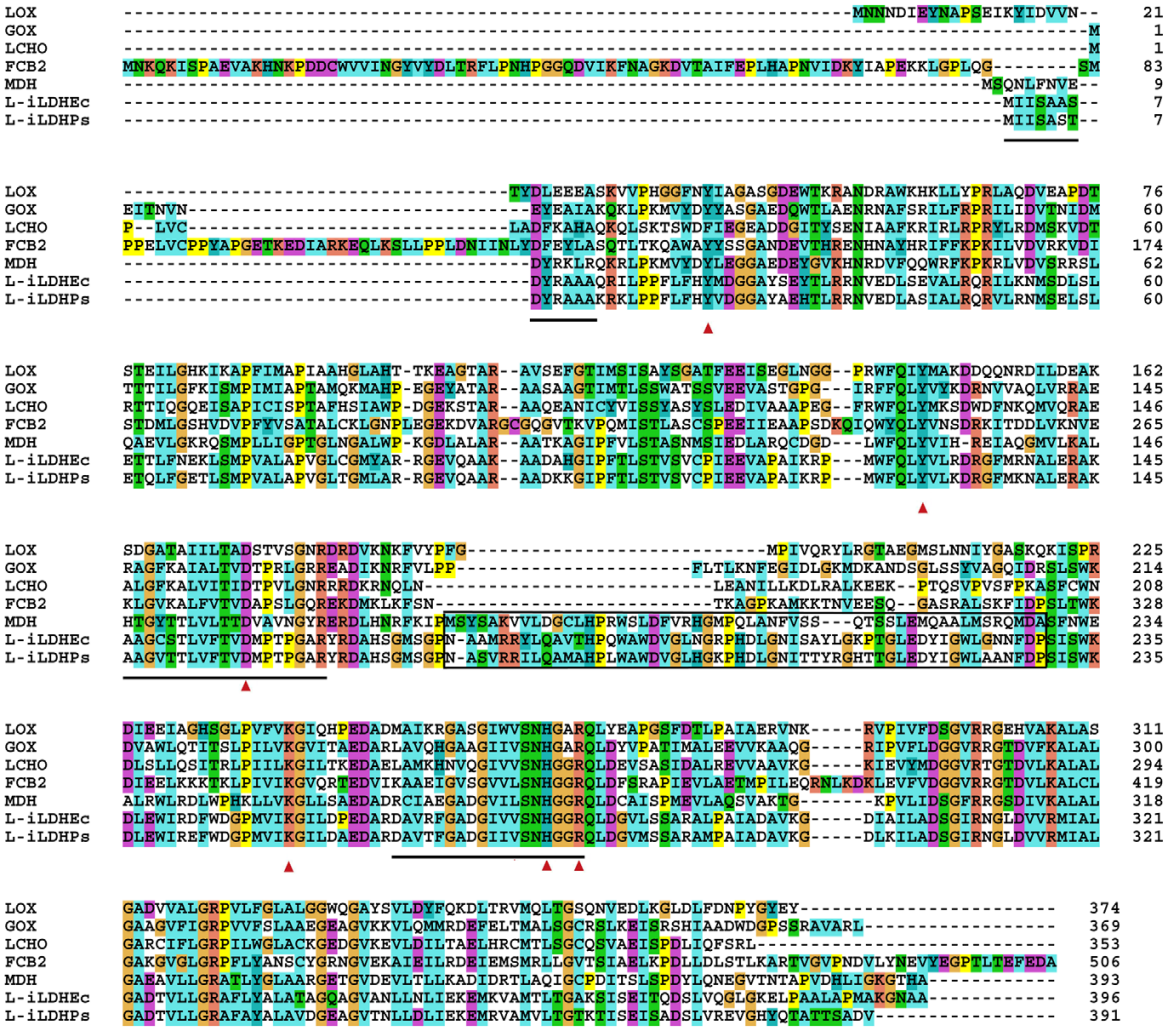
Sequence alignment of l-iLDH from *P. stutzeri* SDM with other FMN-dependent α-hydroxy acid oxidases. LOX, l-lactate oxidase in *A. viridans* [19]; LCHO, long-chain α-hydroxy acid oxidase in rat kidney [20]; MDH, l-mandelate dehydrogenase in *P. putida* [21]; GOX, glycolate oxidase in *S. oleracea* [22]; FCB2, flavocytochrome *b2* (l-iLDH) in *S. cerevisiae* [18]; l-iLDHPs, l-iLDH in *P. stutzeri* SDM; l-iLDHEc, l-iLDH in *E. coli* [9]. Red arrows indicate the highly conserved residues important for FMN binding and enzymatic catalysis across this class of enzymes. The boxed segments in l-mandelate dehydrogenase represent the internal sequences that were implicated in membrane association. The lined segments in l-iLDH in *P. stutzeri* SDM represent the peptides identified through Edman degradation analysis and ESI-MS/MS. The sequences were aligned with the program CLUSTAL X [23]. Parameters and color scheme were set as default.

Considerable sequence identity exists between l-iLDH in *P. stutzeri* SDM and the proteins in the family of the l-α-hydroxyacid-oxidizing flavoproteins, including flavocytochrome *b_2_* in *S. cerevisiae* (29% sequence identity) [18], l-iLDH in *E. coli* (83% sequence identity) [10], l-lactate oxidase in *Aerococcus viridans* (30% sequence identity) [19], long-chain α-hydroxy acid oxidase in rat kidney (32% sequence identity) [20], l-mandelate dehydrogenase in *P. putida* (38% sequence identity) [21], and glycolate oxidase in *Spinacia oleracea* (36% sequence identity) [22]. Based on the resolved crystal structure of flavocytochrome *b*_2_ from *S. cerevisiae*, six conserved amino acid residues required for flavin binding and enzymatic catalysis were identified. As shown in Figure 3, according to bioinformatic analysis with the program CLUSTAL X [23], the residues are highly conserved in all the members of the l-α-hydroxyacid-oxidizing flavoproteins family, including l-iLDH from *P. stutzeri* SDM.

Among the proteins compared in Figure 3, l-mandelate dehydrogenase in *P. putida* and l-iLDHs in *P. stutzeri* SDM and *E. coli* are all membrane-bound proteins [10], [13], [21]. In an earlier study, it was shown that l-mandelate dehydrogenase has an internal insertion which is responsible for membrane association [24], [25]. l-iLDHs from *P. stutzeri* SDM and *E. coli* also have an internal insertion very similar to that found in l-mandelate dehydrogenase, in contrast to the soluble l-α-hydroxyacid-oxidizing flavoproteins (Figure 3). Therefore, like l-mandelate dehydrogenase, l-iLDH from *P. stutzeri* SDM might also be anchored in the membrane through an internal membrane-anchoring segment (Figure 3).

### Properties of l-iLDH

**Figure 4:**
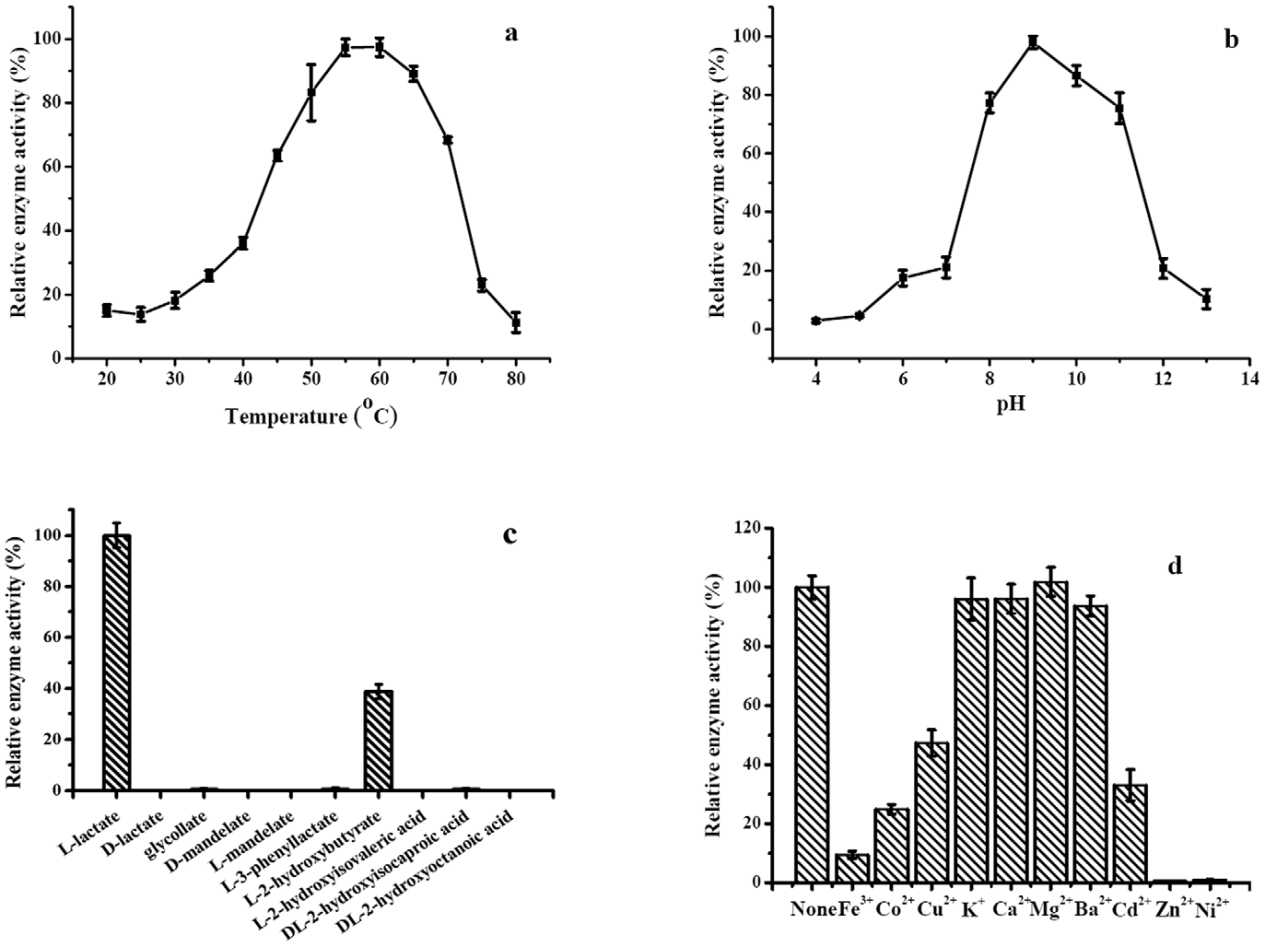
Basic enzymatic properties of l-iLDH from *P. stutzeri* SDM. The reaction mixture contained 20 mM l-lactate, 0.2 mM MTT, 50 mM Tris-HCl (pH 7.5), and 0.1 µg purified l-iLDH. (a) Effect of temperature on l-iLDH activity. Enzyme reactions were carried out at pH 7.5 at different temperatures (20–80°C). (b) Effect of pH on l-iLDH activity. The optimum pH was assessed in a buffer containing citric acid, KH_2_PO_4_, boric acid, and barbital (CKBB buffer) at various pHs (3.0–13.0) at 30°C. (c) Substrate specificity of l-iLDH from *P. stutzeri* SDM. The sodium salts of different α-hydroxy acids were used at a concentration of 20 mM. (d) Effect of different metal ions on l-iLDH activity. The concentration of metal ions was 5 mM in 50 mM Tris-HCl (pH 7.5). Values are the average ± SD of three separate determinations.

l-iLDH activities of various *Pseudomonas* strains have been characterized using the crude cell extract [3]–[5], [13], [14]. l-iLDH was expressed in *E. coli* C43 (DE3) and purified to homogeneity, as described in the “Materials and Methods” (Figure S3). The purified recombinant l-iLDH showed a UV-visible absorption spectrum and cofactor ratio similar to that of the native protein. Effect of temperature on activity of the recombinant l-iLDH in *P. stutzeri* SDM was investigated over a range of 20–80°C; maximum activity was observed at 55°C (Figure 4a). The pH dependence of the recombinant l-iLDH was also investigated over a range of 4.0–13.0. The results showed that l-iLDH exhibited maximum activity at pH 9.0 (Figure 4b).

Substrate specificity of the recombinant l-iLDH from *P. stutzeri* SDM was examined with 20 mM α-hydroxy acids (l-lactate, d-lactate, glycollate, d-mandelate, l-mandelate, l-3-phenyllactate, l-2-hydroxybutyrate, l-2-hydroxyisovaleric acid, dl-2-hydroxyisocaproic acid, dl-2-hydroxyoctanoic acid) and 3-(4,5-dimethylthiazol-2-yl)-2,5-diphenyltetrazolium bromide (MTT) as the electron acceptor (Figure 4c). The *P. stutzeri* SDM l-iLDH seems to have narrow substrate specificity. Only l-lactate and l-2-hydrobutyrate were clearly oxidized by the enzyme. The same results were observed in *E. coli*, implying similar functions of l-iLDHs in l-lactate metabolism of *E. coli* and *P. stutzeri* SDM [9], [10]. In contrast, l-iLDH in *Neisseria gonorrhoeae* exhibited broader substrate specificity, which allowed the strain to catalyze the conversion of l-phenyllactate to phenylpyruvate [26].

The rate of dehydrogenation of l-lactate and l-2-hydrobutyrate catalyzed by the recombinant l-iLDH from *P. stutzeri* SDM followed Michaelis-Menten kinetics. Double-reciprocal plots of the initial rates plotted against the concentrations of l-lactate and l-2-hydroxybutylate were linear at a fixed concentration of MTT (0.2 mM), and yielded *K_m_* values of 29±0.65 μM and 99±3.9 μM, respectively, at 30°C. *V*_max_ was estimated to be 332.3±5.4 μmol·min^−1^·mg^−1^ for l-lactate and 305.4±7.9 μmol·min^−1^·mg^−1^ for l-2-hydroxybutylate with MTT as the electron acceptor (Figure S4). The *K_m_* for l-lactate of l-iLDH in *P. stutzeri* SDM was lower than that of other homologous enzymes like l-iLDH in *E. coli* [10], flavocytochrome *b*_2_ in *S. cerevisiae* [18], and l-lactate oxidase in *A. viridans* [19], in spite of some differences in the experimental conditions (Table S2). Inhibition of l-iLDH by oxalate and oxamate, which are canonical inhibitors of iLDHs [8], was also studied. Both oxalate and oxamate competitively inhibited l-iLDH activity (Figure S5), and their *K*_i_ values were 1.9±0.1 mM and 29±1.7 mM, respectively.

The role of metal ions on activity of the recombinant *P. stutzeri* SDM l-iLDH was tested by adding metal salts (final concentration, 5 mM) to the assay buffer. As shown in Figure 4d, Ca^2+^, K^+^, Mg^2+^, and Ba^2+^ did not affect l-iLDH activity. However, Fe^3+^, Co^2+^, Cu^2+^, and Cd^2+^ partially decreased l-iLDH activity, while Zn^2+^ and Ni^2+^ completely inhibited l-iLDH activity. Experiments carried out with native protein gave similar results (data not shown).

### Kinetic analysis of l-iLDH

**Figure 5:**
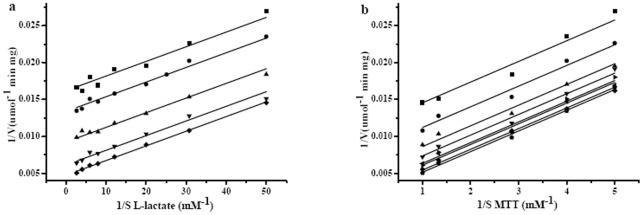
Kinetic mechanism of l-iLDH from *P. stutzeri* SDM. Purified l-iLDH (0.1 µg) was incubated in a reaction mixture containing 50 mM Tris-HCl (pH 7.5) at 30°C. (a) The reaction was started with different l-lactate concentrations at variable MTT concentrations. ▪, 0.2 mM MTT; •, 0.25 mM MTT, ▴, 0.35 mM MTT; ▾, 0.75 mM MTT; ⧫, 0.1 mM MTT. (b) The reaction was started with different MTT concentrations at variable l-lactate concentrations. ▪, 0.02 mM l-lactate; •, 0.0325 mM l-lactate; ▴, 0.05 mM l-lactate; ▾, 0.0825 mM l-lactate; ◂, 0.125 mM l-lactate; ▸, 0.1675 mM l-lactate; ⧫, 0.25 mM l-lactate; <$>\scale 80%\raster="rg1"<$>, 0.375 mM l-lactate.

A set of parallel straight lines was obtained in the double-reciprocal plot of different concentrations of MTT plotted against the activity at fixed concentrations of l-lactate or different concentrations of l-lactate plotted against the activity at fixed concentrations of MTT (Figure 5a, 5b). This kinetic behavior is typical of a double-displacement kinetic (ping-pong) mechanism. A ping-pong kinetic mechanism for l-iLDH in *P. stutzeri* SDM is in agreement with previous reports describing the same mechanism for other l-α-hydroxyacid-oxidizing flavoproteins [19].

### Function of l-iLDH *in vivo*

Many microorganisms can use l-lactate as their sole carbon source for growth [6]–[9], [27]. In these l-lactate utilization processes, l-lactate is first converted to pyruvate, which subsequently enters the tricarboxylic acid cycle [6]–[9], [12], [27]. Thus, the enzymes catalyzing l-lactate into pyruvate are very important for the survival of these microorganisms in habitats containing l-lactate [6]–[9], [12], [27]. In *E. coli* and *Corynebacterium glutamicum*, the l-lactate induced l-iLDHs oxidized l-lactate *in vivo*. Disruption of l-iLDH resulted in the loss of ability to utilize l-lactate as the sole carbon source [9], [28]. Constitutively expressed NAD-dependent l-lactate dehydrogenase would enable the l-iLDH inactivation mutant of *C. glutamicum* to grow on l-lactate [29]. In the present study, l-iLDH from *P. stutzeri* SDM was purified and characterized. The enzyme was able to catalyze the conversion of l-lactate into pyruvate *in vitro* (Figure S6). To identify the function of l-iLDH *in vivo*, insertional inactivation of the l-iLDH encoding gene, *lldD*, in *P. stutzeri* SDM was conducted using the pK18mob system.

We investigated whether the mutant is impaired in growth on solid minimal salt medium (MSM) with l-lactate as the sole carbon source. The mutant *P. stutzeri* SDM-d*lldD* exhibited little growth compared to the wild type (Figure S7a). As a control, both the wild-type and *P. stutzeri* SDM-d*lldD* strains grew equally well on solid MSM with 0.5% pyruvate as the sole carbon source (Figure S7b).

**Figure 6:**
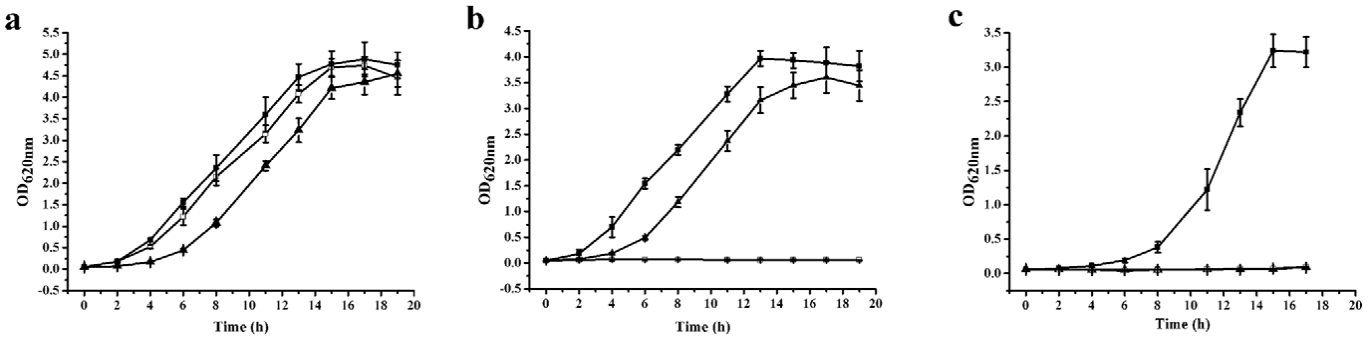
Time-course study of *P. stutzeri* SDM growth on media with (**a**) **pyruvate,** (**b**) **l-lactate, and** (**c**) **d-lactate.** (▪) Wild-type *P. stutzeri* SDM; (□) *P. stutzeri* SDM-d*lldD*; (▴) *P. stutzeri* SDM-d*lldD* harboring the plasmids pBSP II SKlldD. Data are presented as the average ± standard deviation values of 3 parallel replicates.

As with growth on solid MSM, the wild-type and *P. stutzeri* SDM-d*lldD* strains grew well in liquid MSM with 0.5% pyruvate as the sole carbon source (Figure 6a). However, compared with the wild type, *P. stutzeri* SDM-d*lldD* exhibited no growth in liquid MSM with 0.5% l-lactate as the sole carbon source (Figure 6b). Growth in MSM with l-lactate as the sole carbon source could be restored when *P. stutzeri* SDM-d*lldD* were complemented with a broad-host-range plasmid pBSP II SK(–) harboring the *lldD* gene. Altogether, these results suggest that the *lldD* gene encodes l-iLDH, which catalyzes the indispensable transformation of l-lactate to pyruvate in the *P. stutzeri* SDM l-lactate utilization process.

Interestingly, the l-iLDH inactivation mutant of *P. stutzeri* SDM also lost its ability to grow in liquid MSM with 0.5% d-lactate as the sole carbon source. Additionally, the expression of gene *lldD* was not able to complement the phenotype of d-lactate utilization (Figure 6c). In *E. coli* and *C. glutamicum*, the NAD-independent d-lactate dehydrogenase (d-iLDH) that catalyzes d-lactate oxidation *in vivo* was constitutively expressed, while l-iLDH was in the l-lactate utilization operon and controlled by the regulator LldR [9], [28], [30], [31]. In a previous study, d-iLDH activity in *P. stutzeri* SDM was studied. Both l-iLDH and d-iLDH were induced by either enantiomer of lactate in *P. stutzeri* SDM [13]. Because the insertion of pK18mob into the genome of *P. stutzeri* SDM influenced the transcription of the l-iLDH encoding gene and resulted in the inability of *P. stutzeri* SDM to use d-lactate as the sole carbon source, it was hypothesized that the l-iLDH and d-iLDH encoding genes might be in the same operon. Indeed, four adjacent genes (*lldR, lldP, lldD*, and *dld-II*) encoding a lactate-responsive regulator LldR, an l-lactate permease, l-iLDH, and a predicted d-iLDH, were annotated in the draft genome sequence of *P. stutzeri* SDM [32].

Many *Pseudomonas* strains have been isolated as opportunistic human pathogens. *Pseudomonas* infections often elevate human fluid NAD-dependent l-lactate dehydrogenase (l-nLDH) levels [1], [2], [33], [34]. The concentration of l-lactate, the product of l-nLDH-catalyzed pyruvate reduction, might also increase in human fluid during infection. Studies on some microorganisms such as *Neisseria meningitidis* and *N. gonorrhoeae* showed that, in addition to the general stimulation of metabolism, utilization of l-lactate in human hosts also promotes the production of some determinants of pathogenicity [35]–[38]. Thus, the utilization of l-lactate in human hosts has been found to increase the pathogenicity of these organisms [35]. As for the situation in different *Pseudomonas* strains, it has not been determined whether the metabolism of l-lactate would affect their pathogenicity. If a mutant pathogenic *Pseudomonas* strain incapable of utilizing l-lactate is isolated, a comparison of the pathogenicity between the wild-type and mutant strains might provide useful information related to the pathogenic mechanisms of *Pseudomonas* strains.

In summary, l-iLDH in l-lactate utilization strain *P. stutzeri* SDM was purified and further characterized. This key enzyme was confirmed to be required for the l-lactate utilization of strain SDM. Considering the high sequence identity among the *lldD* genes in various *Pseudomonas* strains, l-iLDHs might also play an indispensable function in the l-lactate utilization of other *Pseudomonas* strains.

## Materials and Methods

### Chemicals

l-Lactate, glycollate, d-mandelate, l-mandelate, l-3-phenyllactate, l-2-hydroxybutyrate, l-2-hydroxyisovaleric acid, dl-2-hydroxyisocaproic acid, dl-2-hydroxyoctanoic acid MTT, FMN, bovine serum albumin (BSA), isopropyl-β-d-1-thiogalactopyranoside (IPTG), phenylmethanesulfonyl fluoride (PMSF), and dithiothreitol (DTT) were purchased from Sigma. d-Lactate was purchased from Fluka. All other chemicals were of the reagent grade.

### Bacteria and culture conditions

Bacterial strains used in this study are listed in Table 2. The *P. stutzeri* SDM (CCTCC no: M206010), isolated from soil, was cultured in MSM supplemented with different sole carbon source (10 g·L^−1^) at 30°C [13]. *E. coli* strains were grown in lysogenic broth (LB) medium at 37°C. Antibiotics were used, when appropriate, at the following concentrations: ampicillin (100 μg·mL^−1^), chloramphenicol (50 μg·mL^−1^), and kanamycin (50 μg·mL^−1^).

**Table 2:** The strains used in this work.

Strain	Characteristics a	Source or reference
*P. stutzeri* SDM	Undomesticated wild strain capable of l-Lactate utilizing	[13]
*P. stutzeri* SDM-d*lldD*	Km^r^; *P. stutzeri* SDM mutant obtained by disruption of the *lldD* gene	This study
*P. stutzeri* SDM-d*lldD*(pBSP II SKlldD)	Km^r^; Ap^r^; *P. stutzeri* SDM-d*lldD* harboring the plasmids pBSP II SKlldD	This study
*E. coli* DH5α	F^−^ *Sup*E44, Δ(*arg*F *lac*Zya)U169 ϕ80*lac*ZΔM15 *hsd*R17(r_k_^−^,m_k_^+^) *rec*A1 *end*A1 *gyr*A96 *thi*-1 *rel*A1 λ^−^	Invitrogen
*E. coli* DH10B	F^−^ endA1 recA1 galE15 galK16 nupG rpsL ΔlacX74 Φ80lacZΔM15 araD139 Δ(ara,leu)7697 mcrA Δ(mrr-hsdRMS-mcrBC) λ^−^	Invitrogen
*E. coli* HB101	F^−^ mcrB mrr hsdS20(r_B_^−^ m_B_^−^) recA13 leuB6 ara-14 proA2 lacY1 galK2 xyl-5 mtl-1 rpsL20(Sm^R^) glnV44 λ^−^	Invitrogen
*E. coli* C43 (DE3)	Mutant of *E. coli* BL21 (DE3) for membrane-bound protein expression	[39]

a Km^r^ and Ap^r^ indicate resistance to kanamycin and ampicillin, respectively.

### Purification of l-iLDH

The membrane fraction of *P. stutzeri* SDM was prepared as described by Ma *et* *al*. [13]. Triton X-100 (10%, w/v) was added to the membrane fraction to a final concentration of 1 mg·mg^−1^ protein. The suspensions were stirred gently for 30 min, and then centrifuged at 140,000× *g* for 180 min at 4°C. The supernatants (detergent extracts) were gently transferred into other containers. Solid (NH_4_)_2_SO_4_ was slowly added to the detergent extracts to yield 30% saturation. When (NH_4_)_2_SO_4_ was completely dissolved, the solution was stirred for another 30 min before being centrifuged at 140,000 × *g* for 30 min at 4°C. More (NH_4_)_2_SO_4_ was added to the supernatant to reach 40% saturation, and the solution was stirred and centrifuged as before. The pellet was dissolved in buffer A: 50 mM Tris-HCl (pH 8.6) containing 1 mM DTT, 5 mM MgSO_4_, 0.1% Triton X-100, and 1 mM EDTA, and applied to a column of DEAE Sepharose Fast Flow equilibrated with buffer A. The column was washed with buffer B: 50 mM Tris-HCl (pH 8.6) containing 1 mM DTT, 200 mM KCl, 5 mM MgSO_4_, 0.1% Triton X-100, and 1 mM EDTA, at a flow rate of 5 mL·min^−1^. The fractions containing l-iLDH were concentrated by ultrafiltration and desalted with gel Sephadex G-25. The l-iLDH pool was then applied to a column of SOURCE 30Q pre-equilibrated with buffer A. The column was washed with a linear gradient of 0%–100% buffer B at a flow rate of 5 mL·min^−1^. The fractions containing l-iLDH were concentrated by ultrafiltration and analyzed by SDS-PAGE.

### Analysis of *N*-terminal amino acid and microsequence

The purified l-iLDH was subjected to 12.5% SDS-PAGE for analysis. After electrophoresis, proteins in the gel were electrophoretically transferred to an Immobilon-PSQ PVDF membrane (Millipore) by using 25 mM Tris, 192 mM glycine, and 0.1% SDS as the cathode buffer, and 25 mM Tris, 192 mM glycine, and 20% methanol as the anode buffer. The bands on the PVDF membrane were excised, and sent to the Shanghai GeneCore BioTechnologies Co., Ltd., for *N*-terminal sequencing by automated Edman degradation.

For microsequencing of the internal peptides, the protein bands were cut from SDS-polyacrylamide gels and washed 3 times with water before being digested with trypsin. The resulting peptides were sent to the Shandong Academy of Medical Sciences for ESI-MS/MS.

### Cloning of the l-iLDH encoding gene

The plasmids and primers used in this study are listed in Table 3. Degenerate primers P1, P2, and P3 were constructed according to the N-terminal and internal peptides resulting from the sequencing of *P. stutzeri* SDM l-iLDH. Primer P4 was constructed according to the conserved region of NAD-independent l-lactate dehydrogenase close to l-iLDH in *Pseudomonas* strains. The total genomic DNA of *P. stutzeri* SDM, extracted with the Wizard genomic DNA purification kit (Promega, Madison, WI), was used as the template for the first round of PCR with the primers P1 and P3. One microliter of the PCR products from the reaction mixture was used as the template for the second round of PCR with the primers P1 and P2. Using the genomic DNA of *P. stutzeri* SDM as the template, the third PCR was conducted with the primers P4 and P5. P5 was constructed according to the product of the second round of PCR. After sequencing of the three PCR products, the gene sequence of l-iLDH in *P. stutzeri* SDM was identified (Figure S8).

**Table 3:** The primers and plasmids used in this work.

Plasmid or primer	Characteristics	Source or reference
Plasmid		
pK18mob	Km^r^; *ori*ColE1 Mob^+^, *lacZα*; used for directed insertional disruption	[40]
pK18moblldD	Km^r^; a pK18mob derivative containing an *Bam*HI/*Hin*dIII 322bp internal fragment of the *lldD* gene from *P. stutzeri* SDM	This study
pETDuet-1	Ap^r^; vector for protein expression	Novagen
pET-LDH	pETDuet-1 with *lldD* gene of *P. stutzeri* SDM	This study
pBSP II SK(–)	Ap^r^; broad-host-range expression vector	[41]
pBSP II SKlldD	Ap^r^; pBSP II SK(–) with *lldD* gene of *P. stutzeri* SDM	This study
pRK600	Cm^r^; *ori*ColE1, RK2-Mob^+^, RK2-Tra^+^; helper plasmid for triparental matings	[42]
Primer		
P1	5′- ATGATCATTWSNGCNGC -3′ b	This study
P2	5′- CGGCATRTCSACGGTGAASAC -3′ b	This study
P3	5′- GATGCCRTCSGCGCCGAAGGT -3′ b	This study
P4	5′- GCGCGCCGTCGGCGCGGATTTC -3′	This study
P5	5′- CCAGCATCGCCCTGCGCCAG -3′	This study
Pc1	5′- GGCAAGCTTATGATCATTTCCGCCTCTACC -3′ c	This study
Pc2	5′- ATTCTCGAGTCAGACGTCAGCAGACGTTGT -3′ c	This study
MF	5′- GCGAAGCTTTAACATGTCGGAACTCAGCC -3′ c	This study
MR	5′- ATAGGATCCGGTGGGCATGTCGACGGTGA -3′ c	This study
CF	5′- CCGGTACCATGATCATTTCTGCTTCCACG -3′ c	This study
CR	5′- CTGAGCTCTCAGACGTCAGCAGACGTTGT -3′ c	This study

a Km^r^, Ap^r^, and Cm^r^ indicate resistance to kanamycin, ampicillin, and chloramphenicol, respectively.

b W stands for one of A or T, S stands for one of C or G, R stands for one of A or G, and N stands for A, C, G, or T.

c *Hin*dIII, *Xho*I, *Bam*HI, *Kpn*I and *Sac*I restriction sites introduced in the primers are underlined.

### Purification of recombinant l-iLDH

The l-iLDH encoding gene *lldD*, was amplified by PCR from the genome of *P. stutzeri* SDM by using the primers Pc1 and Pc2. The PCR product was digested by *Xho*I and *Hind*III, and cloned into the *Hind*III/*Xho*I sites of pETDuet-1 to construct the plasmid pET-LDH. *E. coli* C43 (DE3) carrying the pET-LDH plasmid was grown in LB medium (100 μg·mL^−1^ ampicillin) at 37°C to an optical density of 0.6 at 600 nm. Then, 1 mM IPTG was added to induce the expression of l-iLDH. Cells were harvested by centrifugation at 14,000× *g* for 5 min at 4°C and washed with 0.85% (w/v) sodium chloride (NaCl) solution. The cell pellets were subsequently suspended in the binding buffer (pH 7.4, 20 mM sodium phosphate, 20 mM imidazole, 500 mM NaCl, 1 mM PMSF, 0.1% Triton X-100, and 10% glycerol). Approximately 2–2.5 g of wet cells (suspended in 50 mL of binding buffer) were sonicated for 150 cycles (with 5 s at 40% of maximal output and 5 s rest in each cycle) with a Sonics sonicator (500 W/20 KHz, USA) in an ice bath. The sonicate was then centrifuged at 14,000× *g* for 20 min at 4°C. The supernatant was loaded onto a HisTrap HP column (5 mL), and eluted with 80% binding buffer and 20% elution buffer (pH 7.4, 20 mM sodium phosphate, 500 mM imidazole, 500 mM NaCl, 1 mM PMSF, 0.1% Triton X-100, and 10% glycerol) at a flow rate of 5 mL·min^−1^. The fractions containing l-iLDH were concentrated by ultrafiltration, desalted with Sephadex G-25, and then stored in 100 mM sodium phosphate buffer (pH 9.0, containing 50% glycerol and 0.1% Triton X-100) at −20°C. Under these conditions, the enzyme was stable for months with no apparent loss of activity.

### Cofactor analysis of l-iLDH

UV-visible spectra of l-iLDH were recorded at 320–550 nm with an Ultrospec™ 2100 pro UV-visible Spectrophotometer (GE Healthcare Life Sciences). l-iLDH was heated to 100°C for 3 min, and then centrifuged at 10,000 × *g* for 10 min to remove denatured protein. The cofactor released from the purified protein was analyzed by HPLC (Agilent 1100 series; Hewlett-Packard, USA) using an ODS C18 column (4.6×150 mm, particle size: 5 μm) [43]. The eluent was 100 mM ammonium bicarbonate in 82%–18% methanol [43]. Standard FMN solutions of 0.05, 0.1, 0.15, 0.2, 0.25, and 0.3 mM were used for quantitative analysis and detected at 450 nm.

### Knockout of gene *lldD*

To construct the *P. stutzeri* SDM-d*lldD* mutant strains, an internal fragment (322 bp) of the target *lldD* gene was PCR amplified by using the primers MF and MR, which contain *Hin*dIII and *Bam*HI restriction enzyme sites, respectively. The PCR product was cloned into *Hin*dIII/*Bam*HI-cut pK18mob, a mobilizable plasmid that does not replicate in *Pseudomonas*, to form pK18moblldD [44]. To transfer plasmid pK18moblldD into *P. stutzeri* SDM, a triparental filter mating was performed as previously described by using *E. coli* DH10B (pK18moblldD) as the donor strain, *E. coli* HB101 (pRK600) as the helper strain, and *P. stutzeri* SDM as the recipient strain [44]. *P. stutzeri* SDM exconjugants harboring the disrupted *lldD* gene (*P. stutzeri* SDM-d*lldD*) were isolated on minimal medium plates containing citrate that was selected for the *Pseudomonas* recipient cells and kanamycin that was selected for the insertion of the suicide vector after incubation at 30°C for 16 h [44]. The mutant strain was analyzed by PCR to confirm disruption of the target gene (Table S1 and Figure S9).

### Complementation of *P. stutzeri* SDM-d*lldD*

To achieve complementation of the *P. stutzeri* SDM-d*lldD* mutant, the *lldD* gene of *P. stutzeri* SDM was amplified by PCR using the primers CF and CR, which contain *Kpn*I and *Sac*I restriction enzyme sites, respectively. The PCR products were cloned into the *Kpn*I/*Sac*I restriction sites (indicated in the primers) of the vector pBSP II SK(–) [41], which contains an IPTG inducible promoter. The recombinant plasmid pBSP II SKlldD was then introduced into the *P. stutzeri* SDM-d*lldD* mutant by triparental filter mating [44]. The *P. stutzeri* SDM-d*lldD* harboring the plasmid pBSP II SKlldD was isolated on minimal medium plates containing citrate, ampicillin, and kanamycin.

### Growth in MSM

To test bacterial growth on solid media, wild-type and mutant strains were first streaked on LB agar medium and grown at 30°C. The next day, cells from single colonies on LB agar medium were picked and streaked onto solid MSM. Solid MSM was supplemented with l-lactate, d-lactate, or pyruvate as the sole carbon source.

To test bacterial growth in liquid medium, cells were first grown in LB medium until log phase, and then diluted with 20 mL of minimal medium containing l-lactate, d-lactate, or pyruvate as the sole carbon source. Cells were then grown at 30°C with shaking. IPTG was added at a final concentration of 1 mM to the minimal media. At various time points, the cell densities (OD_620nm_) of the cultures were recorded with an Ultrospec™ 2100 pro UV/visible spectrophotometer.

### Biochemical assays

The activity of l-iLDH was determined at 30°C in 1 mL of 50 mM Tris-HCl (pH 7.5) and 0.2 mM MTT. The reaction was started by addition of l-lactate, and the rate of MTT reduction was determined by measuring the changes in absorbance at 578 nm [45]. One unit of l-iLDH activity was defined as the amount reducing 1.0 μmol of electron acceptor per minute under the test conditions. Protein concentrations were determined by the Lowry method, with BSA as the standard [46].

### Polyacrylamide gel electrophoresis

SDS-PAGE was performed using a 12.5% polyacrylamide resolving gel and a 4% polyacrylamide stacking gel. Electrophoresis was run at a constant voltage of 80 V when the samples were in the stacking gel. When the dye front reached the resolving gel, the voltage was increased to 120 V. The run was stopped when the dye front was 2–3 mm away from the bottom edge of the gel.

### Nucleotide sequence accession number

The nucleotide sequence of the *lldD* gene has been deposited in the GenBank nucleotide sequence databases under the accession no. GU373722.

## Supporting Information

Figure S1 **HPLC analysis of** (**a**) **authentic FMN, and** (**b**) **the cofactor released from the purified l-iLDH in** ***P. stutzeri*** **SDM.** For the identification of the cofactor of l-iLDH, the purified l-iLDH was heated to 100°C for 3 min and then centrifuged at 10,000× g for 10 min to remove denatured protein. Cofactor released from purified protein was analyzed by HPLC (Agilent 1100 series, Hewlett-Packard, USA) using an ODS C18 column (4.6×150 mm, particle size: 5 μm). The eluent was 100 mM ammonium bicarbonate 82–18% methanol. As shown in Figure S1, a compound identical to authentic FMN was produced. Therefore, l-iLDH in *P. stutzeri* SDM used FMN as the cofactor.
(PDF)

Figure S2 **Standard curve of FMN concentration related to peak area obtained by HPLC analysis.** For the determination of the ratio between l-iLDH and FMN, 0.1171 mM l-iLDH was heated to 100°C for 3 min and then centrifuged at 10,000× g for 10 min to remove denatured protein. Cofactor released from purified protein was analyzed by HPLC (Agilent 1100 series, Hewlett-Packard, USA) using an ODS C18 column (4.6×150 mm, particle size: 5 μm). The eluent was 100 mM ammonium bicarbonate 82–18% methanol. Standard FMN solutions of 0.05, 0.1, 0.15, 0.2, 0.25, and 0.3 mM were used for quantitative analysis and detected at 450 nm. The concentration of the cofactor released from purified protein was determined to be 0.1136 mM. Thus, the ratio between l-iLDH and FMN was 0.1171/0.1136 = 1.03. Therefore, the native enzyme contains one FMN per subunit.
(PDF)

Figure S3 **SDS-PAGE analysis of over-expression and purification of l-iLDH.** Lane M, molecular weight markers; lane 1, whole cell proteins of *E. coli* C43(DE3) (pET-LDH); lane 2, crude extract of *E. coli* C43(DE3) (pET-LDH); 3, the purified recombinant L-iLDH.
(PDF)

Figure S4 **Apparent** ***K_m_*** **of l-lactate and l-2-hydroxybutylate for l-iLDH in** ***P. stutzeri*** **SDM.** The reaction mixture contained 0.2 mM MTT, 50 mM Tris–HCl (pH 7.5), and 0.1 μg purified l-iLDH. The reaction was started with variable l-lactate and l-2-hydroxybutylate concentrations. Double-reciprocal plots of the initial rates versus the concentration of l-lactate (a) and l-2-hydroxybutylate (b) were linear and yielded  =  the *K*_m_ values of 29±0.65 μM and 99±3.9 μM, respectively, at 30°C. The *V*_max_ values were estimated to be 332.3±5.4 μmol min^−1^ mg^−1^ for l-lactate, and 305.4±7.9 μmol min^−1^ mg^−1^ for l-2-hydroxybutylate, with MTT as the electron acceptor. The dispersion values indicate the standard errors of the mean (SEM) of the linear regression analysis of one experiment.
(PDF)

Figure S5 **Inhibition of l-iLDH by oxalate and oxamate.** Purified l-iLDH (0.1 μg) was incubated in the reaction mixture contained 0.2 mM MTT and 50 mM Tris–HCl (pH 7.5) at 30°C. (a) The reaction was started with different l-lactate concentrations at variable oxalate concentrations. ▪, no oxalate; •, 1.25 mM oxalate; ▴, 2.5 mM oxalate; ▾, 3.75 mM oxalate; ◂, 5 mM oxalate. (b) The reaction was started with different l-lactate concentrations at variable oxamate concentrations. ▪, no oxamate; •, 2.5 mM oxamate; ▴, 5 mM oxamate; ▾, 20 mM oxamate; ◂, 31.25 mM oxamate. The patterns of double-reciprocal plots indicate a competitive inhibition for both chemicals. The *K*_i_ values of oxalate and oxamate were estimated to be 1.9±0.1 mM and 29±1.7 mM, respectively. The dispersion values indicate the SEM of the linear regression analysis of one experiment.
(PDF)

Figure S6 **HPLC analysis of the product of the reaction catalyzed by l-iLDH.** (a), authentic l-lactate; (b), authentic pyruvate; (c) the reaction mixture after 4 h. To determine the l-lactate oxidation product of the l-iLDH in *P. stutzeri* SDM, biotransformation was carried out using l-lactate (10 mM) and MTT (5 mM) as the substrate and purified l-iLDH as the biocatalyst in 50 mM Tris–HCl (pH 7.5) at 30°C. After 4 h of reaction, the mixture was centrifuged at 20,000× g for 15 min. The supernatant was analyzed by a HPLC system (Agilent 1100 series, Hewlett-Packard, USA) equipped with an Aminex HPX-87H column (Bio-Rad). The mobile phase (10 mM H_2_SO_4_) was pumped at 0.4 ml min^−1^ (55°C). As shown in Figure S6c, a compound identical to authentic pyruvate was produced. Similar to other reported l-iLDH, the l-iLDH in *P. stutzeri* SDM also catalyzes the conversion of l-lactate into pyruvate.
(PDF)

Figure S7 **The** ***lldD*** **gene is required for growth on l-lactate.** (a), growth of wild-type and mutant strains of *P. stutzeri* SDM on solid minimal media containing 0.5% pyruvate as the sole carbon source. (b), growth of the same set of strains (shown in panels a) on solid minimal media containing 0.5% l-lactate as the sole carbon source. We constructed the *P. stutzeri* SDM mutants lacking the *lldD*. Whether the mutants were impaired in growth on solid minimal medium with 0.5% l-lactate as the sole carbon source was tested. As shown in Figure S7b, the mutant exhibited little growth compared to the wild type. As a control, both the wild type and the mutant grew equally well on solid minimal medium with 0.5% pyruvate as the sole carbon source (Figure S7a).
(PDF)

Figure S8 **Scheme for the** ***lldD*** **gene cloning procedure.**
(PDF)

Figure S9 **PCR analysis for verification of the insertional inactivation of the gene encoding l-iLDH. the l-iLDH encoding gene** ***lldD*** **.** (A) Structure of pK18moblldD (left), the l-iLDH encoding gene *lldD* in *P. stutzeri* SDM genome (right), the insertional inactivated l-iLDH encoding gene *lldD* in *P. stutzeri* SDM genome (bottom). (B) PCR verification of the mutant strains (Figure S7) with the insertion of pK18mob in *P. stutzeri* SDM genome. In all cases, the primer pair VF1/VR1 (arrows; for the sequence, see Table S1) was used. (C) PCR verification of the mutant strains (Figure S7) with the homologus recombination between pK18moblldD and *P. stutzeri* SDM genome. In all cases, the primer pair VF2/VR2 (arrows; for the sequence, see Table S1) was used.
(PDF)

Table S1 **Primers used in the verification of the insertional inactivation of the l-iLDH encoding gene.**
(DOC)

Table S2 **Comparison of** ***K*** **_m_ values estimated for different enzymes.**
(DOC)
