# Supplementary material for: NAD-Independent L-Lactate Dehydrogenase Is Required for L-Lactate Utilization in Pseudomonas stutzeri SDM
Source: PLoS One. 2012 May 4;7(5):e36519. doi: 10.1371/journal.pone.0036519 (PMC3344892; doi:10.1371/journal.pone.0036519)
Supplement: Figure S1 — HPLC analysis of (a) authentic FMN, and (b) the cofactor released from the purified l-iLDH in P. stutzeri SDM. For the identification of the cofactor of l-iLDH, the purified l-iLDH was heated to 100°C for 3 min and then centrifuged at 10,000× g for 10 min to remove denatured protein. Cofactor released from purified protein was analyzed by HPLC (Agilent 1100 series, Hewlett-Packard, USA) using an ODS C18 column (4.6×150 mm, particle size: 5 μm). The eluent was 100 mM ammonium bicarbonate 82–18% methanol. As shown in Figure S1, a compound identical to authentic FMN was produced. Therefore, l-iLDH in P. stutzeri SDM used FMN as the cofactor. (PDF) [file pone.0036519.s001.pdf]

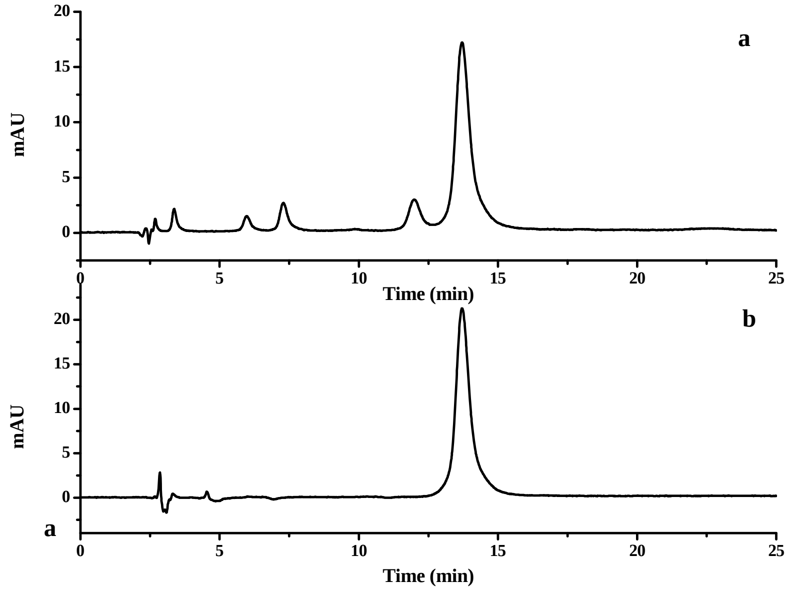

Figure S1. HPLC analysis of (a) authentic FMN, and (b) the cofactor released from the purified L-iLDH in *P. stutzeri* SDM. For the identification of the cofactor of L-iLDH, the purified L-iLDH was heated to 100°C for 3 min and then centrifuged at 10,000 × g for 10 min to remove denatured protein. Cofactor released from purified protein was analyzed by HPLC (Agilent 1100 series, Hewlett-Packard, USA) using an ODS C18 column (4.6 × 150 mm, particle size: 5 μm). The eluent was 100 mM ammonium bicarbonate 82-18% methanol. As shown in Figure S1, a compound identical to authentic FMN was produced. Therefore, L-iLDH in *P. stutzeri* SDM used FMN as the cofactor.
