# Supplementary material for: NAD-Independent L-Lactate Dehydrogenase Is Required for L-Lactate Utilization in Pseudomonas stutzeri SDM
Source: PLoS One. 2012 May 4;7(5):e36519. doi: 10.1371/journal.pone.0036519 (PMC3344892; doi:10.1371/journal.pone.0036519)
Supplement: Figure S2 — Standard curve of FMN concentration related to peak area obtained by HPLC analysis. For the determination of the ratio between l-iLDH and FMN, 0.1171 mM l-iLDH was heated to 100°C for 3 min and then centrifuged at 10,000× g for 10 min to remove denatured protein. Cofactor released from purified protein was analyzed by HPLC (Agilent 1100 series, Hewlett-Packard, USA) using an ODS C18 column (4.6×150 mm, particle size: 5 μm). The eluent was 100 mM ammonium bicarbonate 82–18% methanol. Standard FMN solutions of 0.05, 0.1, 0.15, 0.2, 0.25, and 0.3 mM were used for quantitative analysis and detected at 450 nm. The concentration of the cofactor released from purified protein was determined to be 0.1136 mM. Thus, the ratio between l-iLDH and FMN was 0.1171/0.1136 = 1.03. Therefore, the native enzyme contains one FMN per subunit. (PDF) [file pone.0036519.s002.pdf]

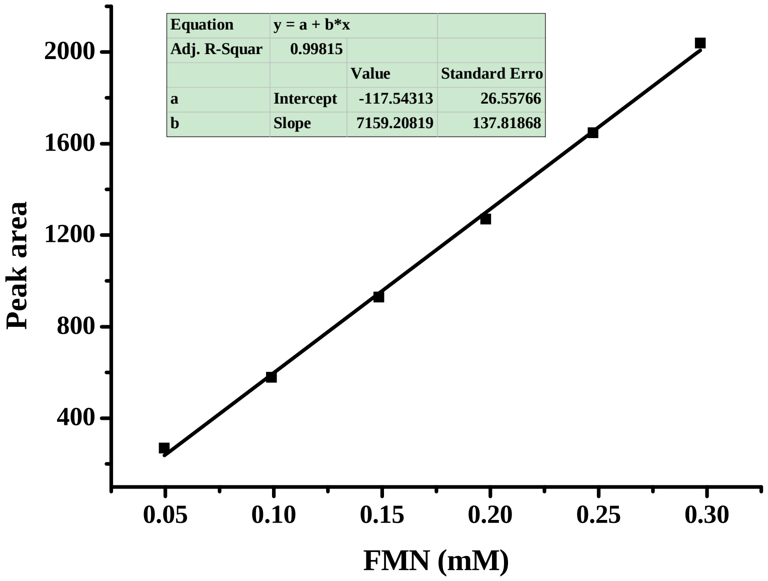

Figure S2. Standard curve of FMN concentration related to peak area obtained by HPLC analysis. For the determination of the ratio between L-iLDH and FMN, 0.1171 mM L-iLDH was heated to 100°C for 3 min and then centrifuged at 10,000 × g for 10 min to remove denatured protein. Cofactor released from purified protein was analyzed by HPLC (Agilent 1100 series, Hewlett-Packard, USA) using an ODS C18 column (4.6 × 150 mm, particle size: 5 μm). The eluent was 100 mM ammonium bicarbonate 82-18% methanol. Standard FMN solutions of 0.05, 0.1, 0.15, 0.2, 0.25, and 0.3 mM were used for quantitative analysis and detected at 450 nm. The concentration of the cofactor released from purified protein was determined to be 0.1136 mM. Thus, the ratio between L-iLDH and FMN was 0.1171/0.1136=1.03. Therefore, the native enzyme contains one FMN per subunit.
