# Supplementary material for: NAD-Independent L-Lactate Dehydrogenase Is Required for L-Lactate Utilization in Pseudomonas stutzeri SDM
Source: PLoS One. 2012 May 4;7(5):e36519. doi: 10.1371/journal.pone.0036519 (PMC3344892; doi:10.1371/journal.pone.0036519)
Supplement: Figure S3 — SDS-PAGE analysis of over-expression and purification of l-iLDH. Lane M, molecular weight markers; lane 1, whole cell proteins of E. coli C43(DE3) (pET-LDH); lane 2, crude extract of E. coli C43(DE3) (pET-LDH); 3, the purified recombinant L-iLDH. (PDF) [file pone.0036519.s003.pdf]

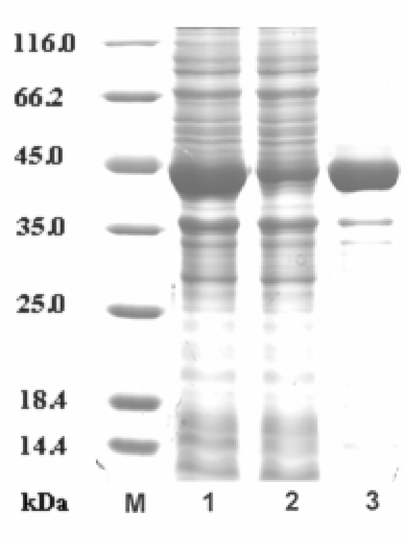

Figure S3. SDS-PAGE analysis of over-expression and purification of L-iLDH. Lane M, molecular weight markers; lane 1, whole cell proteins of *E. coli* C43(DE3) (pET-LDH); lane 2, crude extract of *E. coli* C43(DE3) (pET-LDH); 3, the purified recombinant L-iLDH.
