# Supplementary material for: NAD-Independent L-Lactate Dehydrogenase Is Required for L-Lactate Utilization in Pseudomonas stutzeri SDM
Source: PLoS One. 2012 May 4;7(5):e36519. doi: 10.1371/journal.pone.0036519 (PMC3344892; doi:10.1371/journal.pone.0036519)
Supplement: Figure S4 — Apparent Km of l-lactate and l-2-hydroxybutylate for l-iLDH in P. stutzeri SDM. The reaction mixture contained 0.2 mM MTT, 50 mM Tris–HCl (pH 7.5), and 0.1 μg purified l-iLDH. The reaction was started with variable l-lactate and l-2-hydroxybutylate concentrations. Double-reciprocal plots of the initial rates versus the concentration of l-lactate (a) and l-2-hydroxybutylate (b) were linear and yielded = the K m values of 29±0.65 μM and 99±3.9 μM, respectively, at 30°C. The V max values were estimated to be 332.3±5.4 μmol min−1 mg−1 for l-lactate, and 305.4±7.9 μmol min−1 mg−1 for l-2-hydroxybutylate, with MTT as the electron acceptor. The dispersion values indicate the standard errors of the mean (SEM) of the linear regression analysis of one experiment. (PDF) [file pone.0036519.s004.pdf]

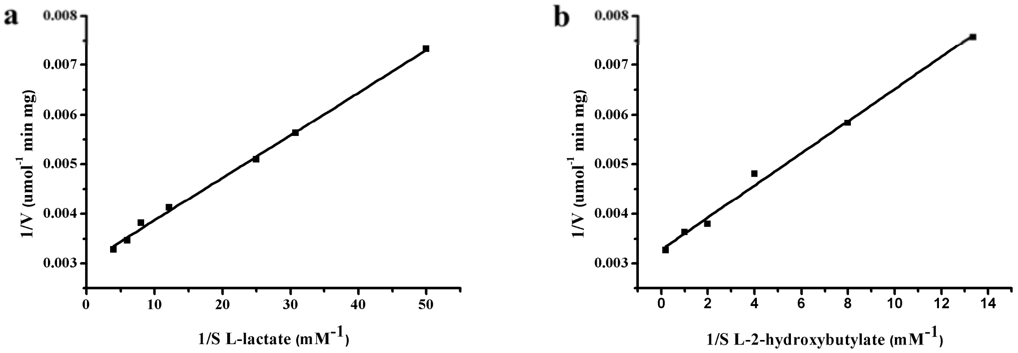


**Figure S4. Apparent $K_m$ of L-lactate and L-2-hydroxybutylate for L-iLDH in *P. stutzeri* SDM.** The reaction mixture contained 0.2 mM MTT, 50 mM Tris–HCl (pH 7.5), and 0.1 μg purified L-iLDH. The reaction was started with variable L-lactate and L-2-hydroxybutylate concentrations. Double-reciprocal plots of the initial rates versus the concentration of L-lactate (a) and L-2-hydroxybutylate (b) were linear and yielded the $K_m$ values of 29 ± 0.65 μM and 99 ± 3.9 μM, respectively, at 30°C. The $V_{max}$ values were estimated to be 332.3 ± 5.4 μmol $min^{-1}$ $mg^{-1}$ for L-lactate, and 305.4 ± 7.9 μmol $min^{-1}$ $mg^{-1}$ for L-2-hydroxybutylate, with MTT as the electron acceptor. The dispersion values indicate the standard errors of the mean (SEM) of the linear regression analysis of one experiment.
