# Supplementary material for: NAD-Independent L-Lactate Dehydrogenase Is Required for L-Lactate Utilization in Pseudomonas stutzeri SDM
Source: PLoS One. 2012 May 4;7(5):e36519. doi: 10.1371/journal.pone.0036519 (PMC3344892; doi:10.1371/journal.pone.0036519)
Supplement: Figure S5 — Inhibition of l-iLDH by oxalate and oxamate. Purified l-iLDH (0.1 μg) was incubated in the reaction mixture contained 0.2 mM MTT and 50 mM Tris–HCl (pH 7.5) at 30°C. (a) The reaction was started with different l-lactate concentrations at variable oxalate concentrations. ▪, no oxalate; •, 1.25 mM oxalate; ▴, 2.5 mM oxalate; ▾, 3.75 mM oxalate; ◂, 5 mM oxalate. (b) The reaction was started with different l-lactate concentrations at variable oxamate concentrations. ▪, no oxamate; •, 2.5 mM oxamate; ▴, 5 mM oxamate; ▾, 20 mM oxamate; ◂, 31.25 mM oxamate. The patterns of double-reciprocal plots indicate a competitive inhibition for both chemicals. The K i values of oxalate and oxamate were estimated to be 1.9±0.1 mM and 29±1.7 mM, respectively. The dispersion values indicate the SEM of the linear regression analysis of one experiment. (PDF) [file pone.0036519.s005.pdf]

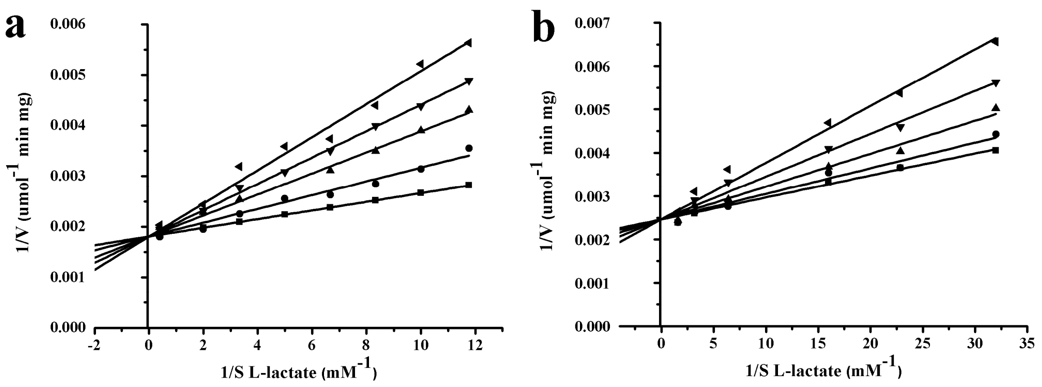

Figure S5. Inhibition of L-iLDH by oxalate and oxamate. Purified L-iLDH (0.1 μg) was incubated in the reaction mixture contained 0.2 mM MTT and 50 mM Tris–HCl (pH 7.5) at 30°C. (a) The reaction was started with different L-lactate concentrations at variable oxalate concentrations. ■, no oxalate; ●, 1.25 mM oxalate; ▲, 2.5 mM oxalate; ▼, 3.75 mM oxalate; ◄, 5 mM oxalate. (b) The reaction was started with different L-lactate concentrations at variable oxamate concentrations. ■, no oxamate; ●, 2.5 mM oxamate; ▲, 5 mM oxamate; ▼, 20 mM oxamate; ◄, 31.25 mM oxamate. The patterns of double-reciprocal plots indicate a competitive inhibition for both chemicals. The K_i values of oxalate and oxamate were estimated to be 1.9 ± 0.1 mM and 29 ± 1.7 mM, respectively. The dispersion values indicate the SEM of the linear regression analysis of one experiment.
