# Supplementary material for: NAD-Independent L-Lactate Dehydrogenase Is Required for L-Lactate Utilization in Pseudomonas stutzeri SDM
Source: PLoS One. 2012 May 4;7(5):e36519. doi: 10.1371/journal.pone.0036519 (PMC3344892; doi:10.1371/journal.pone.0036519)
Supplement: Figure S6 — HPLC analysis of the product of the reaction catalyzed by l-iLDH. (a), authentic l-lactate; (b), authentic pyruvate; (c) the reaction mixture after 4 h. To determine the l-lactate oxidation product of the l-iLDH in P. stutzeri SDM, biotransformation was carried out using l-lactate (10 mM) and MTT (5 mM) as the substrate and purified l-iLDH as the biocatalyst in 50 mM Tris–HCl (pH 7.5) at 30°C. After 4 h of reaction, the mixture was centrifuged at 20,000× g for 15 min. The supernatant was analyzed by a HPLC system (Agilent 1100 series, Hewlett-Packard, USA) equipped with an Aminex HPX-87H column (Bio-Rad). The mobile phase (10 mM H2SO4) was pumped at 0.4 ml min−1 (55°C). As shown in Figure S6c, a compound identical to authentic pyruvate was produced. Similar to other reported l-iLDH, the l-iLDH in P. stutzeri SDM also catalyzes the conversion of l-lactate into pyruvate. (PDF) [file pone.0036519.s006.pdf]

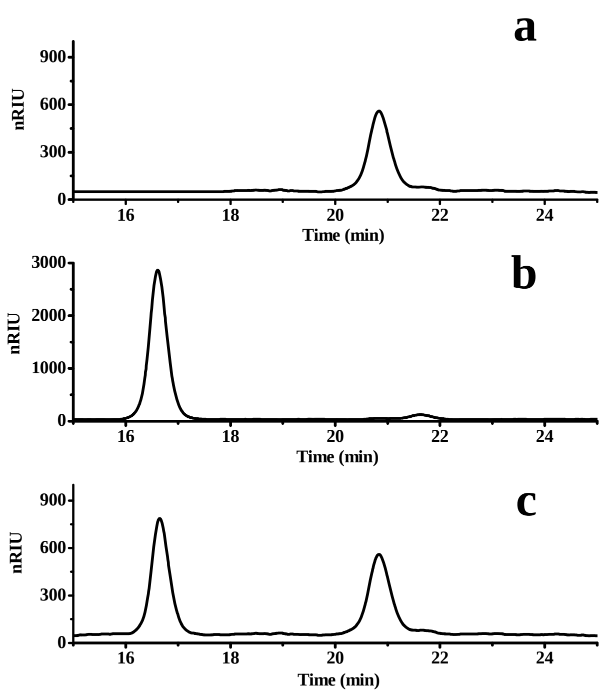

Figure S6. HPLC analysis of the product of the reaction catalyzed by L-iLDH. (a), authentic L-lactate; (b), authentic pyruvate; (c) the reaction mixture after 4 h. To determine the L-lactate oxidation product of the L-iLDH in *P. stutzeri* SDM, biotransformation was carried out using L-lactate (10 mM) and MTT (5 mM) as the substrate and purified L-iLDH as the biocatalyst in 50 mM Tris–HCl (pH 7.5) at 30°C. After 4 h of reaction, the mixture was centrifuged at 20,000 × g for 15 min. The supernatant was analyzed by a HPLC system (Agilent 1100 series, Hewlett-Packard, USA) equipped with an Aminex HPX-87H column (Bio-Rad). The mobile phase (10 mM H_2SO_4) was pumped at 0.4 ml min^{-1} (55°C). As shown in Figure S6c, a compound identical to authentic pyruvate was produced. Similar to other reported L-iLDH, the L-iLDH in *P. stutzeri* SDM also catalyzes the conversion of L-lactate into pyruvate.
