# Supplementary material for: NAD-Independent L-Lactate Dehydrogenase Is Required for L-Lactate Utilization in Pseudomonas stutzeri SDM
Source: PLoS One. 2012 May 4;7(5):e36519. doi: 10.1371/journal.pone.0036519 (PMC3344892; doi:10.1371/journal.pone.0036519)
Supplement: Figure S7 — The lldD gene is required for growth on l-lactate. (a), growth of wild-type and mutant strains of P. stutzeri SDM on solid minimal media containing 0.5% pyruvate as the sole carbon source. (b), growth of the same set of strains (shown in panels a) on solid minimal media containing 0.5% l-lactate as the sole carbon source. We constructed the P. stutzeri SDM mutants lacking the lldD. Whether the mutants were impaired in growth on solid minimal medium with 0.5% l-lactate as the sole carbon source was tested. As shown in Figure S7b, the mutant exhibited little growth compared to the wild type. As a control, both the wild type and the mutant grew equally well on solid minimal medium with 0.5% pyruvate as the sole carbon source (Figure S7a). (PDF) [file pone.0036519.s007.pdf]

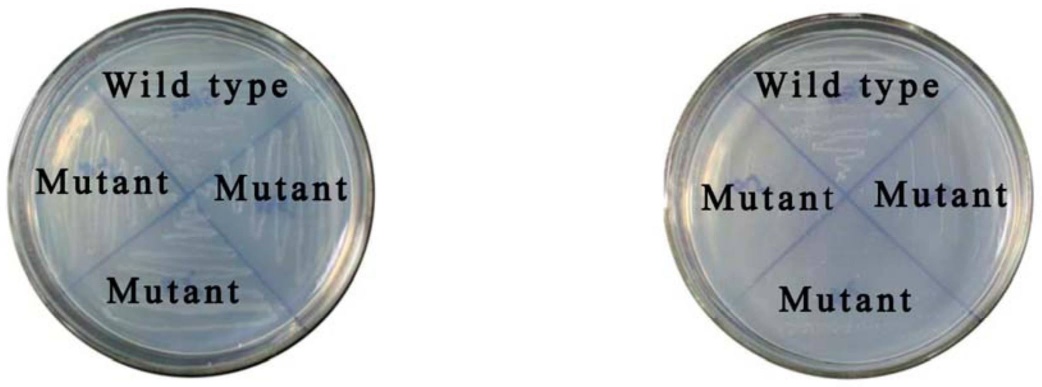

(a) (b)

Figure S7. The *lldD* gene is required for growth on L-lactate. (a), growth of wild-type and mutant strains of *P. stutzeri* SDM on solid minimal media containing 0.5% pyruvate as the sole carbon source. (b), growth of the same set of strains (shown in panels a) on solid minimal media containing 0.5% L-lactate as the sole carbon source. We constructed the *P. stutzeri* SDM mutants lacking the *lldD*. Whether the mutants were impaired in growth on solid minimal medium with 0.5% L-lactate as the sole carbon source was tested. As shown in Figure S7b, the mutant exhibited little growth compared to the wild type. As a control, both the wild type and the mutant grew equally well on solid minimal medium with 0.5% pyruvate as the sole carbon source (Figure S7a).
