# Supplementary figures and images for: NAD-Independent L-Lactate Dehydrogenase Is Required for L-Lactate Utilization in Pseudomonas stutzeri SDM
Source: PLoS One. 2012 May 4;7(5):e36519. doi: 10.1371/journal.pone.0036519 (PMC3344892; doi:10.1371/journal.pone.0036519)

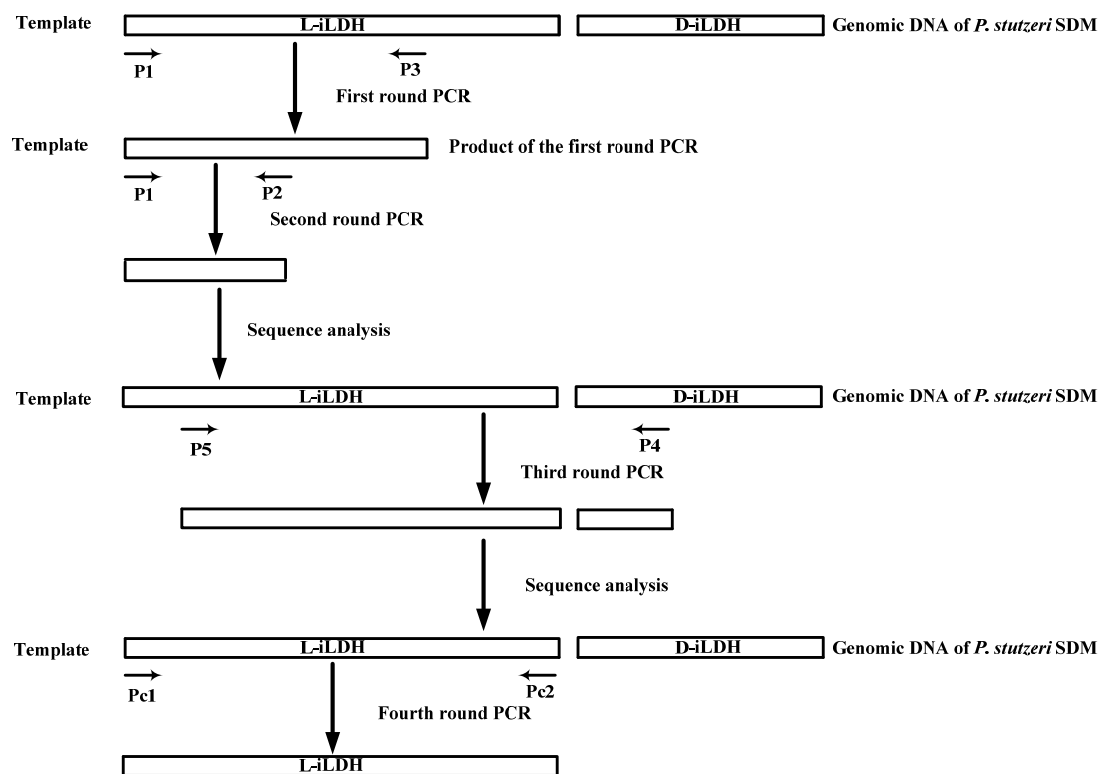

**Figure S8.** The scheme for the L-iLDH gene cloning procedure.

Supplement: Figure S8 — Scheme for the lldD gene cloning procedure. (PDF) [file pone.0036519.s008.pdf]
