# Supplementary material for: NAD-Independent L-Lactate Dehydrogenase Is Required for L-Lactate Utilization in Pseudomonas stutzeri SDM
Source: PLoS One. 2012 May 4;7(5):e36519. doi: 10.1371/journal.pone.0036519 (PMC3344892; doi:10.1371/journal.pone.0036519)
Supplement: Figure S9 — PCR analysis for verification of the insertional inactivation of the gene encoding l-iLDH. the l-iLDH encoding gene lldD . (A) Structure of pK18moblldD (left), the l-iLDH encoding gene lldD in P. stutzeri SDM genome (right), the insertional inactivated l-iLDH encoding gene lldD in P. stutzeri SDM genome (bottom). (B) PCR verification of the mutant strains (Figure S7) with the insertion of pK18mob in P. stutzeri SDM genome. In all cases, the primer pair VF1/VR1 (arrows; for the sequence, see Table S1) was used. (C) PCR verification of the mutant strains (Figure S7) with the homologus recombination between pK18moblldD and P. stutzeri SDM genome. In all cases, the primer pair VF2/VR2 (arrows; for the sequence, see Table S1) was used. (PDF) [file pone.0036519.s009.pdf]

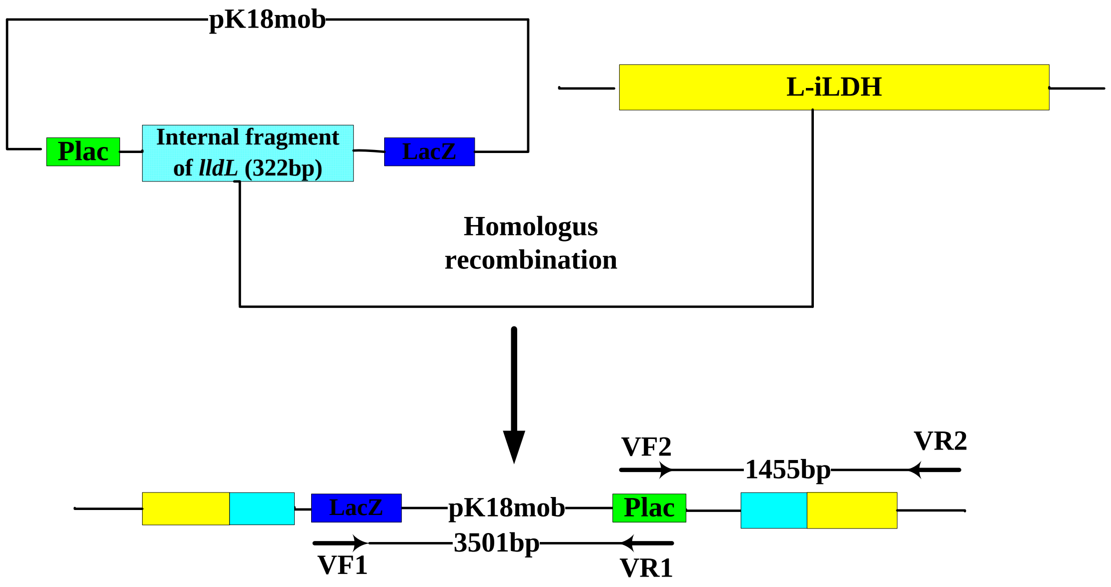

(A)

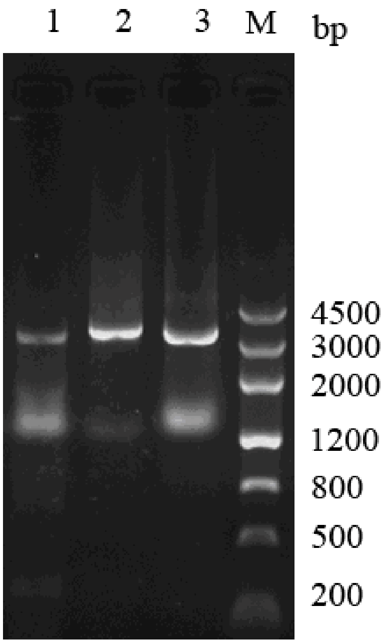

(B)

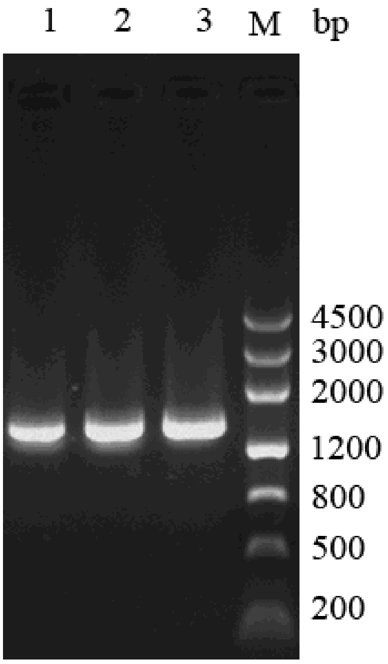

(C)

Figure S9. PCR analysis for verification of the insertional inactivation of the gene encoding L-iLDH. the L-iLDH encoding gene *lldD*. (A) Structure of pK18moblldD (left), the L-iLDH encoding gene *lldD* in *P. stutzeri* SDM genome (right), the insertional

inactivated L-iLDH encoding gene *lldD* in *P. stutzeri* SDM genome (bottom). (B) PCR verification of the mutant strains (Figure S7) with the insertion of pK18mob in *P. stutzeri* SDM genome. In all cases, the primer pair VF1/VR1 (arrows; for the sequence, see Table S1) was used. (C) PCR verification of the mutant strains (Figure S7) with the homologus recombination between pK18moblldD and *P. stutzeri* SDM genome. In all cases, the primer pair VF2/VR2 (arrows; for the sequence, see Table S1) was used.
