# Supplementary material for: NAD-Independent L-Lactate Dehydrogenase Is Required for L-Lactate Utilization in Pseudomonas stutzeri SDM
Source: PLoS One. 2012 May 4;7(5):e36519. doi: 10.1371/journal.pone.0036519 (PMC3344892; doi:10.1371/journal.pone.0036519)
Supplement: Table S1 — Primers used in the verification of the insertional inactivation of the l-iLDH encoding gene. (DOC) [file pone.0036519.s010.doc]

Table S1. Primers used in the verification of the insertional inactivation of the l-iLDH encoding gene

| Description | Sequence |
| --- | --- |
| VF1 | 5'- TCCACACAACATACGAGCCG -3' |
| VR1 | 5'- TCACGCTGCCGCAAGCACTC -3' |
| VF2 | 5'- GCTTCCCAACCTTACCAGAG -3' |
| VR2 | 5'- TCAGACGTCAGCAGACGTTG -3' |
