# Supplementary material for: NAD-Independent L-Lactate Dehydrogenase Is Required for L-Lactate Utilization in Pseudomonas stutzeri SDM
Source: PLoS One. 2012 May 4;7(5):e36519. doi: 10.1371/journal.pone.0036519 (PMC3344892; doi:10.1371/journal.pone.0036519)
Supplement: Table S2 — Comparison of K m values estimated for different enzymes. (DOC) [file pone.0036519.s011.doc]

Table S2. Comparison of *K*_m_ values estimated for different enzymes

| Taxon | Enzyme | *K*_m_ (mM) | Buffer composition | Buffer pH | Temp (°C) | Electron acceptor | Reference |
| --- | --- | --- | --- | --- | --- | --- | --- |
| *P. stutzeri* | l-iLDH | 0.029 | Tris–HCl | 7.5 | 30 | MTT | This study |
| *E. coli* | l-iLDH | 0.12 | Tris–HCl | 8.0 | 23 | Tetrazolium | 10 |
| *S. cerevisiae* | Flavocytochrome *b*_2_ | 0.49 | Tris–HCl | 7.5 | 25 | Ferricyanide | 18 |
| *A. viridans* | l-Lactate oxidase | 0.94 | Imidazole | 7.0 | 25 | O_2_ | 19 |
